# A 10-year case study on the changing determinants of university student satisfaction in the UK

**DOI:** 10.1371/journal.pone.0192976

**Published:** 2018-02-23

**Authors:** Adrian Burgess, Carl Senior, Elisabeth Moores

**Affiliations:** 1 Department of Psychology, Aston University, Birmingham, United Kingdom; 2 University of Gibraltar, Gibraltar, Gibraltar; CPERI, GREECE

## Abstract

Higher Education (HE), once the prerogative of a tiny elite, is now accessible to larger numbers of people around the world than ever before yet despite the fact that an understanding of student satisfaction has never been more important for today’s universities, the concept remains poorly understood. Here we use published data from the UK’s National Student Survey (NSS), representing data from 2.3 million full-time students collected from 2007 to 2016, as a case study of the benefits and limitations of measuring student satisfaction that might have applicability for other countries, particularly those that, like the UK, have experienced significant growth in student numbers. The analyses showed that the factor structure of the NSS remained generally stable and that the ability of the NSS to discriminate between different subjects at different universities actually improved over the ten-year sample period. The best predictors of overall satisfaction were ‘Teaching Quality’ and ‘Organisation & Management’, with ‘Assessment & Feedback’ having relatively weak predictive ability, despite the sector’s tangible efforts to improve on this metric. The tripling of student fees in 2012 for English students (but not the rest of the UK) was used as a ‘natural experiment’ to investigate the sensitivity of student satisfaction ratings to the real economic costs of HE. The tuition fee increase had no identifiable negative effect, with student satisfaction steadily improving throughout the decade. Although the NSS was never designed to measure perceived value-for-money, its insensitivity to major changes in the economic costs of HE to the individual suggest that the conventional concept of student satisfaction is incomplete. As such we propose that the concept of student satisfaction: (i) needs to be widened to take into account the broader economic benefits to the individual student by including measures of perceived value-for-money and (ii) should measure students’ level of satisfaction in the years post-graduation, by which time they may have a greater appreciation of the value of their degree in the workplace.

## Introduction

There is no doubt that Higher Education (HE) brings many advantages to the graduating student as well as a wider range of societal benefits that subsequently drive economic growth [1–3]. The average graduate, for example, can expect to enjoy lifetime earnings far greater than their non-graduate contemporaries (the so-called ‘Graduate Premium) [4–6] so it is no surprise that the global HE sector remains vibrant and that more and more people are applying to study at universities than ever before [7].

This growth of the global HE sector has produced severe challenges to the traditional concept of a university. The model of universities as bastions of an elite liberal education is being replaced across the globe by one in which universities are seen as accessible providers of applicable and relevant knowledge [8–10]. This transition, however, has sometimes had undesirable and unexpected consequences and, with more and more money being spent on HE internationally, the debate has intensified over whether it is actually worth it or not, and more specifically whether it actually delivers social mobility [11–14]. There is evidence, for example, suggest that the growth of HE in China may have actually intensified social inequalities, at least in some circumstances [15]. In addition, in countries where HE has been primarily funded by the state, the growth of the university sector has produced strains on the public purse that have increasingly been seen as unsustainable.

In the UK, state funding meant that university education was free between 1962 and 1998 and underwent considerable growth in that period with participation rates in HE growing from around 7% of all school leavers in 1962 to more than 30% by the late 1990s [16]. From that time onwards, successive governments, persuaded of the economic benefits of further growth in HE, but alarmed at the increasing costs, began to transfer the cost from the state on to individual students with the introduction of tuition fees of £1,000 per year across the UK in 1998. However, as education is a devolved power, tuition fees in the constituent parts of the UK since that time have diverged. The full history of changes in tuition fees is complicated and further details are available in the Supporting Information (S1 File). In summary however, from 2006, students domiciled in England, Northern Ireland and Wales were charged up to £3,000 per annum until 2012 when the maximum fee in England was dramatically increased to £9,000 (increased to £9,250 in 2017/18). Students from Northern Ireland studying at Northern Irish HEIs, experienced a much smaller increase with fees being capped at £3,805 from 2012 onwards. In Wales too, the fee increase was modest and was capped at £4,045 (and this cap applied wherever they attended university in the UK). Only in Scotland, was university education free throughout this period (to Scottish domiciled students only). The UK HE sector, therefore provides a natural experiment on the effect of charging fees on student satisfaction as there is a three-tier system of tuition fees in place: no fee (Scotland), intermediate fees (Wales and Northern Ireland) and high fees (England).

In England, the introduction of ‘variable’ (but capped) tuition fees was intended to create market competition or ‘choice’ between Higher Education Institutions (HEIs) and thereby reduce costs, but this largely failed because the vast majority of English HEIs charged the maximum allowable fee. Indeed, there was a widespread belief that charging less than the maximum fee would be a marker of a low quality that would have a potentially detrimental effect on student enrolment for any course or university that did so [17]. Consequently, in a further push towards marketization (or *“Plan B”* [12], p.1651), the UK government announced that from 2013 Universities in the UK would no longer be restricted in the numbers of students that they could recruit [18]. This decision led to a rapid expansion and increased competition between universities for students. So much so that in 2015, the Competition and Markets Authority (CMA; the branch of the UK Legislature responsible for the protection of markets) required all Universities to comply with consumer protection law. One of the main outcomes of the CMA legislation on student expectations is that they started to be considered consumers in their learning [19].

It is increasingly being recognized that the massification and marketization of HE provision can only be successful if appropriate quality assurance mechanisms are in place to ensure that the learning experience not only remains excellent, but is also applicable and relevant to students’ post-graduation aspirations [20]. There is some debate over whether the quality of education has been affected by expansion [21–23] and over how to define ‘quality’ (e.g., [24]). Implementation of robust quality assurance mechanisms is not a trivial process, but it is one that will have significant impact on the ability of HEIs to meet the needs and expectations of its students. Meaningful and timely measures of student satisfaction need to be available to institutional managers and wider governing bodies to help identify areas of weaknesses. However, there is little doubt that the concept and measurement of student satisfaction is one that vexes institutional managers around the world because, despite its importance, the accurate measurement of this ephemeral entity presents a myriad of challenges.

Across the global HE sector, institutional managers collect information on student satisfaction using a range of mechanisms. These mechanisms are designed to ensure that the expectations of the student are met at every stage of their progression through university and that they contribute effectively to the ongoing governance of the institution. Most developed countries employ some form of national survey that is delivered to the students to collect a range of measures of student satisfaction. These include the Japanese College Student Survey (JCSS) and the Japanese Freshman Survey (JFS), the USA’s National Survey for Student Engagement (NSSE) and Australia’s Course Experience Questionnaire (CEQ) and similar measures deployed to collect information from undergraduate students across the globe [25–28].

In the UK, the annual measurement of student satisfaction is carried out via the administration of the National Student Survey (NSS: [29]), a survey that also supports the position of UK HEIs in various league tables. This is an anonymous student satisfaction survey that was designed along the similar principles to the Australian CEQ [26] and is completed by all final year undergraduate students of publicly (and some privately) funded HEIs, which represents an annual census of nearly 500,000 students. Arguably the two dominant purposes of the collection of such data are to: (i) help Universities assess and improve their student experience and (ii) inform prospective students in making their enrolment decisions [30]. Such information is vitally important to ensure that a particular university remains competitive in the wider market. The results are highly influential and, as Rodgers, Freeman Williams and Kane (2011)[31] state, *"Without doubt*, *the NSS has been very influential in spurring institutions into action that they may never have taken before"* (p.248). However, despite the unquestionable influence of such student satisfaction measures, there is considerable debate as to whether they actually offer sufficient discrimination between universities to be useful, or measure fairly across different subject disciplines [32, 33]. Indeed, it has been argued that the NSS has “*acquired significance that far outweighs its validity or intended use*” [34].

The transformation of the English HE sector towards an open-market has been guided, in part, by student satisfaction provided by the NSS. However, there is a relative paucity of research into the factors which contribute to successful performance in the NSS and into the extent to which the information gained from the NSS *is* actually useful to students when choosing their university and degree programme. Moreover, although the NSS *has* been reviewed and revised for content and purpose [30], there has been no large scale interrogation of the resulting twelve years of data to investigate whether issues of importance to students in 2005, as represented by the factors in the survey, remained relevant in 2016. A long-term study was commissioned by the Higher Education Funding Council of England (HEFCE) who showed that the NSS had a stable factor structure from 2005 to 2013, yet also revealed a slight dip in some of the correlations between questions between 2008 and 2009. It is argued that this dip may have been due to the 2009 population including graduates who had paid the larger £3,000 fees, introduced in 2006. However, the first main cohort of graduates paying £9,000 tuition fees did not graduate until 2015, outside of the scope of the HEFCE analysis. It seems reasonable to speculate that the trebling of tuition fees in 2012, in particular coupled with the rapid expansion of student numbers, might affect overall student satisfaction as well as the factors that determine it. First, any ‘over-supply’ of graduates would mean that students’ degrees would effectively be devalued in the employment marketplace. Second, university resources may have been stretched by the increases in student numbers and third, contemporaneously graduates would have paid more for their degree. Indeed, while early studies that did not use the NSS showed that the first introduction of fees in 1998 to the UK HE sector had a negative effect on overall student satisfaction scores, this was moderated by a number of other factors such as the research culture of particular university [35].

The aim of this paper is to address four critical questions about student satisfaction in the modern world of mass, marketized higher education. First, how well has the NSS worked as a tool for **helping universities improve** by adapting to the demands of their students over the last ten years? Success would be shown by improving student satisfaction scores over the decade, particularly in those areas where student satisfaction was lowest. Second, how useful has the NSS been over the last 10 years in **helping prospective students choose** the best university for their course of choice? Success would be indicated by a higher proportion of variance in NSS scores being attributable to university subject group than to the either the university or subject alone. Third, what have been the best **predictors of overall student satisfaction** over the decade? For example, which aspects of the student experience, as measured by the NSS, have the biggest influence on student satisfaction and have they changed? Finally, is there any evidence that student satisfaction has been affected by the increasing marketization of UK HE sector? Specifically, what have been **the effects of increases in tuition fees** during the period had any measurable effect on student satisfaction?

## Methods

### Description of the data

Data were the published NSS results from 2007 and 2016. These are third party data and were not collected by the authors nor owned by them and the authors did not have any special access privileges to the data. The data are freely available for download from the HEFCE website (http://www.hefce.ac.uk/lt/nss/results/). The NSS consists of 21 items divided into 6 subscales (‘Teaching Quality’, ‘Assessment & Feedback’, ‘Academic Support’, ‘Organisation & Management’, ‘Learning Resources’ and ‘Personal Development’) plus an additional rating of ‘Overall Satisfaction’. HEFCE provides data for multiple categories of students (Full/Part-time, First degree/Other Undergraduate) but here only full-time students studying for their first undergraduate degree were considered. Responses from individual students are not available. Instead, data are in the form of the percentage of students who endorsed responses 1 to 5 (1 = ‘strongly disagree’, 2 = ‘disagree’, 3 = ‘neither agree nor disagree’, 4 = ‘agree’ and 5 = ‘strongly agree’) for each of the 22 NSS questions for each university subject group. For example, 92% of final year students studying psychology at Aston University in 2010 agreed (i.e. responded ‘agree’ or ‘strongly agree’) with the item ‘Overall, I am satisfied with the quality of the course’. Confidence intervals for the percentage of students who endorsed each item are also provided as well as the number of students eligible to take part in the NSS and the number who actually did so.

Between 2007 and 2016, more than 3.6 million students, attending 266 separate HE institutions, were eligible to complete the NSS and 2.5 million (68.9%) actually did so. As the aim was to evaluate the NSS over the decade from 2007 to 2016, only institutions that returned data for every year during that period were included. In addition, although the NSS includes many small HE providers, in this study only the largest 100 HE providers based on student sample size were considered (see S1 Table). These universities, the ‘Big 100’, included 90.3% of eligible students during the period of interest.

The key dependent variable used was the mean percentage agreement on each item of the NSS calculated as the number of students who rated ‘Mostly Agree’ or ‘Definitely Agree’ as a percentage of the total number of responders, excluding those who responded ‘not applicable’. Mean percentage agreement estimates were produced for each of the 4,455 university subject groups (including all 108 NSS Level 3 subject areas—see S2 Table) in the Big 100 universities for each year 2007–2016 that it was available from a median sample size of 50 students (range 10 to 1,349). However, 61% of the subject groups had missing data for one or more years during the decade (median number of years of complete data = 9; iqr. 5 to 10) giving a total of 33,265 subject group mean percentage agreement estimates out of a possible 44,550 (75%).

Of the 100 universities included in this study, 85 were in England, 2 in Northern Ireland, 7 in Scotland and 6 in Wales. Although precise figures for the proportion of students attending English, Northern Irish, Scottish and Welsh universities paying the different levels of tuition fees are not readily available, data on the domicile of students attending university in different parts of the UK are [36]. These suggest that 97.8% of students at English universities pay the higher level of tuition fees, 80% of those at university in Wales or Northern Ireland pay reduced fees and 87% of students at Scottish universities pay no tuition fees. Consequently, by examining NSS scores across the decade in the universities from the different parts of the UK, we can investigate whether the tripling of tuition fees in 2012 had any effect on student satisfaction.

Most HEIs in the UK are affiliated to one of four groups similar to the Ivy League classification in the US. These are the Russell Group (which includes the largest and internationally best known UK HEIs and is research intensive), the 1994 Group (now disbanded, but included smaller research-intensive HEIs), the University Alliance (which includes HEIs with a specific focus on the training of technical and professional skills), and the Million+ group, includes HEIs with a predominant focus on teaching.

### Statistical analysis

#### Sources of variance in NSS scores

In order to determine what role the university attended, subject studied and individual NSS items played in NSS mean satisfaction scores, a Variance Component Analysis was performed. Percentage agreement for each NSS item for each university subject group was the dependent variable with ‘Subject’ (the subject studied, defined with the 108 NSS level 3 subject categories) and ‘University’ (the university attended with a separate code for each of the 100 universities included in the sample) as random factors and ‘NSS item’ (items 1 to 22) as a fixed factor. Variance component analysis of NSS scores was by minimum norm quadratic unbiased estimation (MINQUE) and ANOVA methods in SPSS (v21). As the ANOVA and MINQUE methods gave very similar results, only the MINQUE results are reported here.

#### Predictors of overall satisfaction

To determine which NSS questions most contributed to ‘Overall Satisfaction’, factor analysis was used to reduce the 21 NSS questions to six orthogonal factor scores that mapped onto the six NSS subscales (‘Teaching Quality’, ‘Assessment & Feedback’, ‘Academic Support’, ‘Organisation & Management’, ‘Learning Resources’ and ‘Personal Development’). Factor analysis was by Principal Axis Factoring (PAF) of the 21 NSS items (excluding ‘Overall Satisfaction’) with varimax rotation. The six-factor model accounted for 69.7% of the variance and was consistent with the NSS allocation of items into their subscales (S3 Table) except that item 11, *‘I have been able to contact staff when I needed to’* loaded approximately equally on both ‘Academic Support’ (where it is supposed to belong) and ‘Organisation & Management’, a finding that supports previous analyses [37]. Linear regression in SPSS (v21) was used to identify those NSS factors, derived from the factor analysis, that most contributed to ‘Overall Satisfaction’.

## Results

### Helping universities improve

**Fig 1:**
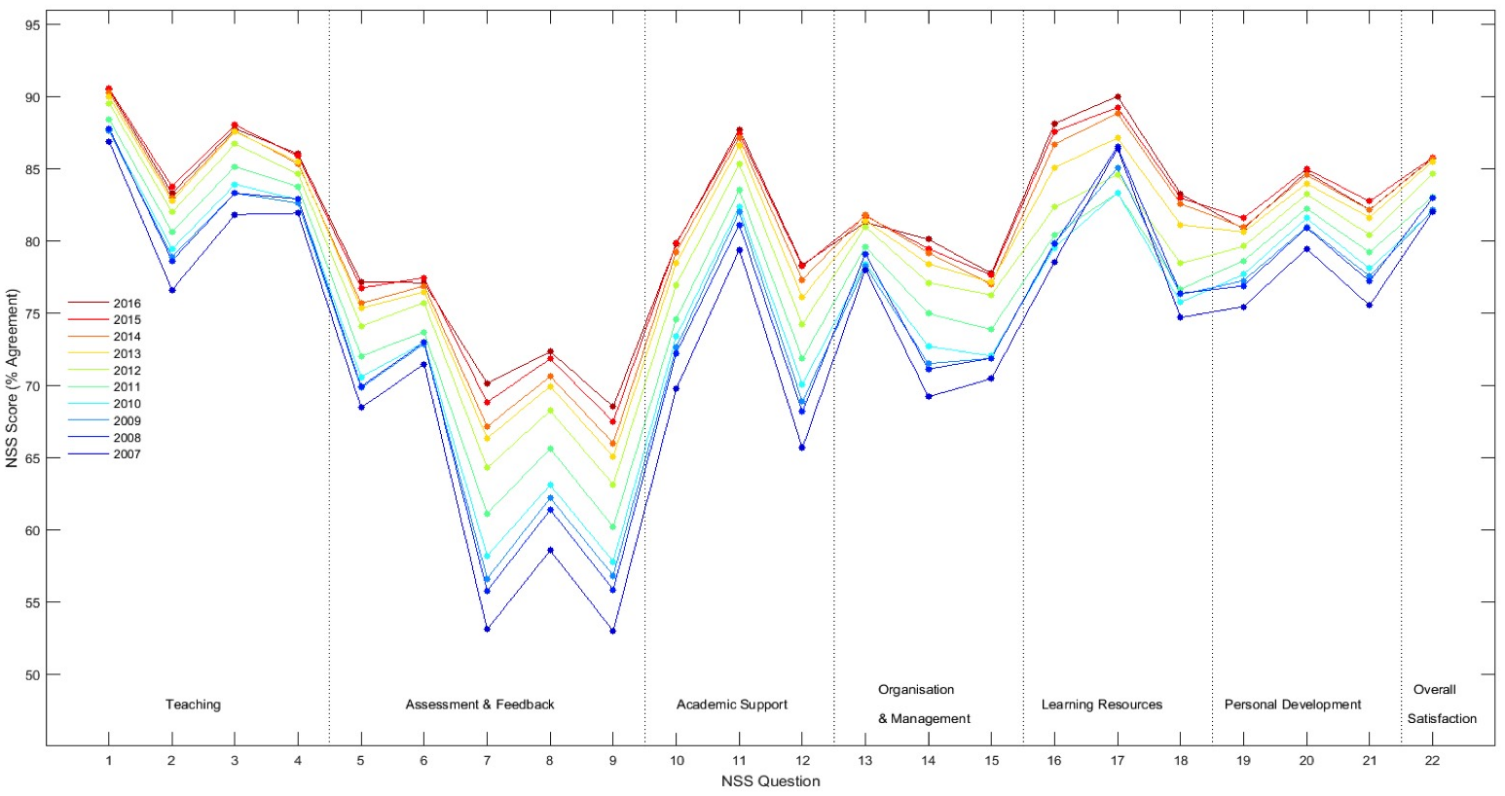
Mean student satisfaction measured using the NSS from 2007 to 2016 (averaged over items 1 to 22) in the UK national cohort.

There has been a remarkable increase in NSS scores for satisfaction over the decade 2007–2016 with the mean agreement for nearly every item showing a consistent increase each year (see Fig 1). Those items that received the lowest rates of agreement at the start of the period (notably Questions 7, 8, 9 & 12, relating to feedback and advice) have shown the greatest improvement. The result is that the profile of scores has tended to both increase and flatten. However, the rate of increase now appears to be slowing, suggesting that NSS scores for many items, including ‘Overall Satisfaction’ are nearing their ceiling.

### Helping prospective students choose

**Fig 2:**
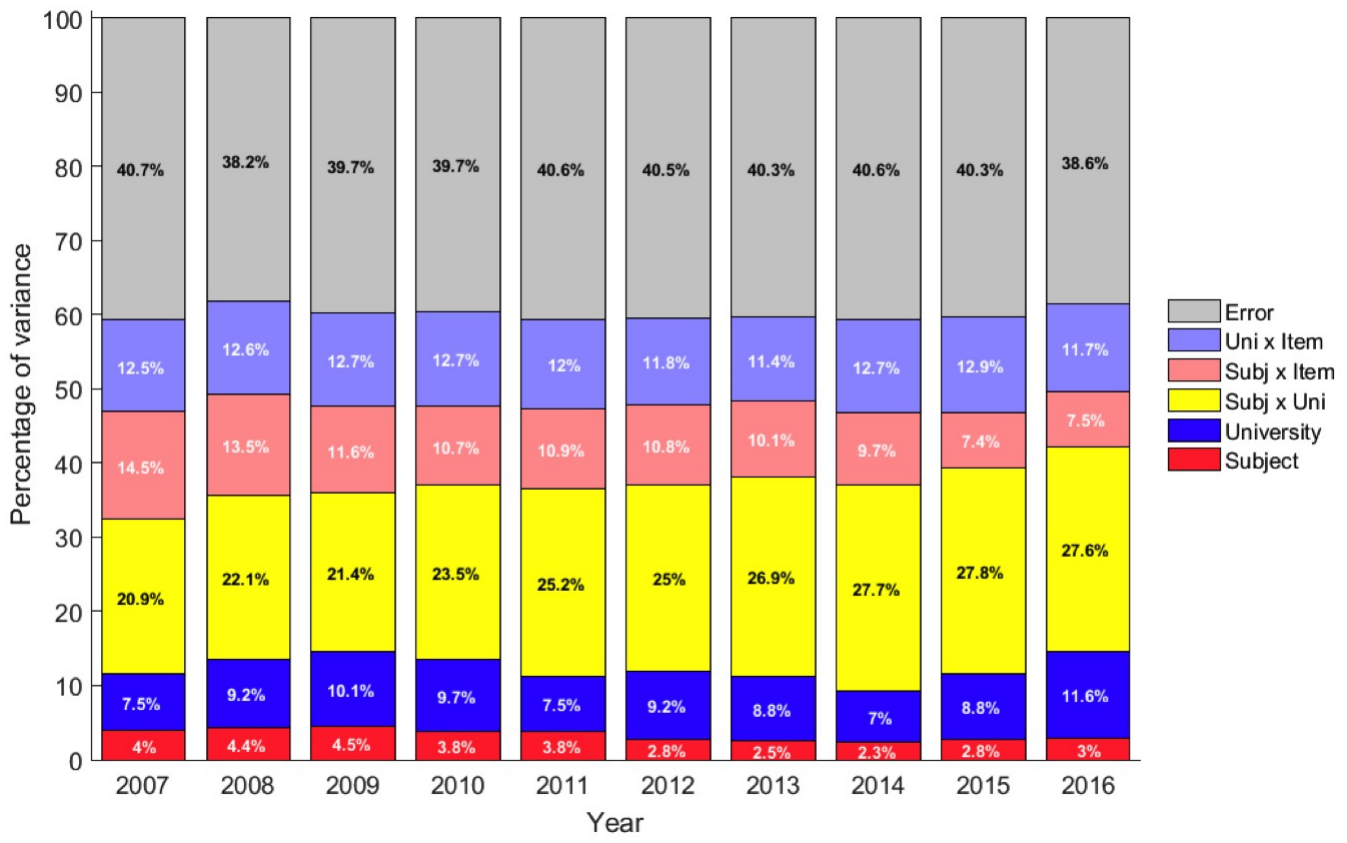
The percentage of variance in NSS scores associated with the subject studied (Subject) and the university attended (University) and their interactions (see Methods for details) for each year from 2007 to 2016.

One of the main objectives of the NSS is to provide prospective students with information to help them choose the best university for the subject they wish to study, so a relatively high proportion of the variance should be attributable to the ‘Subject x University’ interaction, showing that the university is discriminating between courses at different universities. Similarly, the proportion of variance attributable to ‘Subject’ and ‘University’ individually should each be small. As shown in Fig 2, the contribution of ‘University’ to variance in NSS items was modest (7.5% to 11.6%); mean scores over the decade ranged from 68.1% (University of the Arts) to 84.3% (Loughborough University). The contribution of ‘Subject’ to variance in NSS items was even smaller (2.3% to 4.5%), with mean scores ranging from 74.5% (Social Work) to 82.1% (History).

The ‘Subject x University’ interaction was much larger than either the ‘University’ or ‘Subject’ contributions (20.9% to 27.6%), with scores for specific subjects at specific Universities ranging from 99.1 (‘Sociology’ at University College London) to 49.1 (‘Others in European Languages’ at Buckinghamshire New University). The relatively large impact of the ‘Subject x University’ interaction compared with the modest contributions of ‘Subject’ and ‘University’ individually, shows that the NSS is working as its designers might have hoped. Nevertheless, error was the largest source of variance in each year, meaning that most variance was simply unaccounted for.

Over the course of the decade, most of the sources of variance were relatively stable with the exception of the ‘Subject x University’ interaction which showed a steady increase from 20.9% in 2007 to 27.6% in 2016, suggesting that the proportion of variance in NSS scores that is helpful to students choosing the right university for their subject of choice has increased substantially. This increase has been achieved at the cost of the ‘Subject x Item’ interaction which fell from 14.5% to 7.5% in the same period which suggests that the profiles of NSS scores for different subjects have become more similar over time.

**Fig 3:**
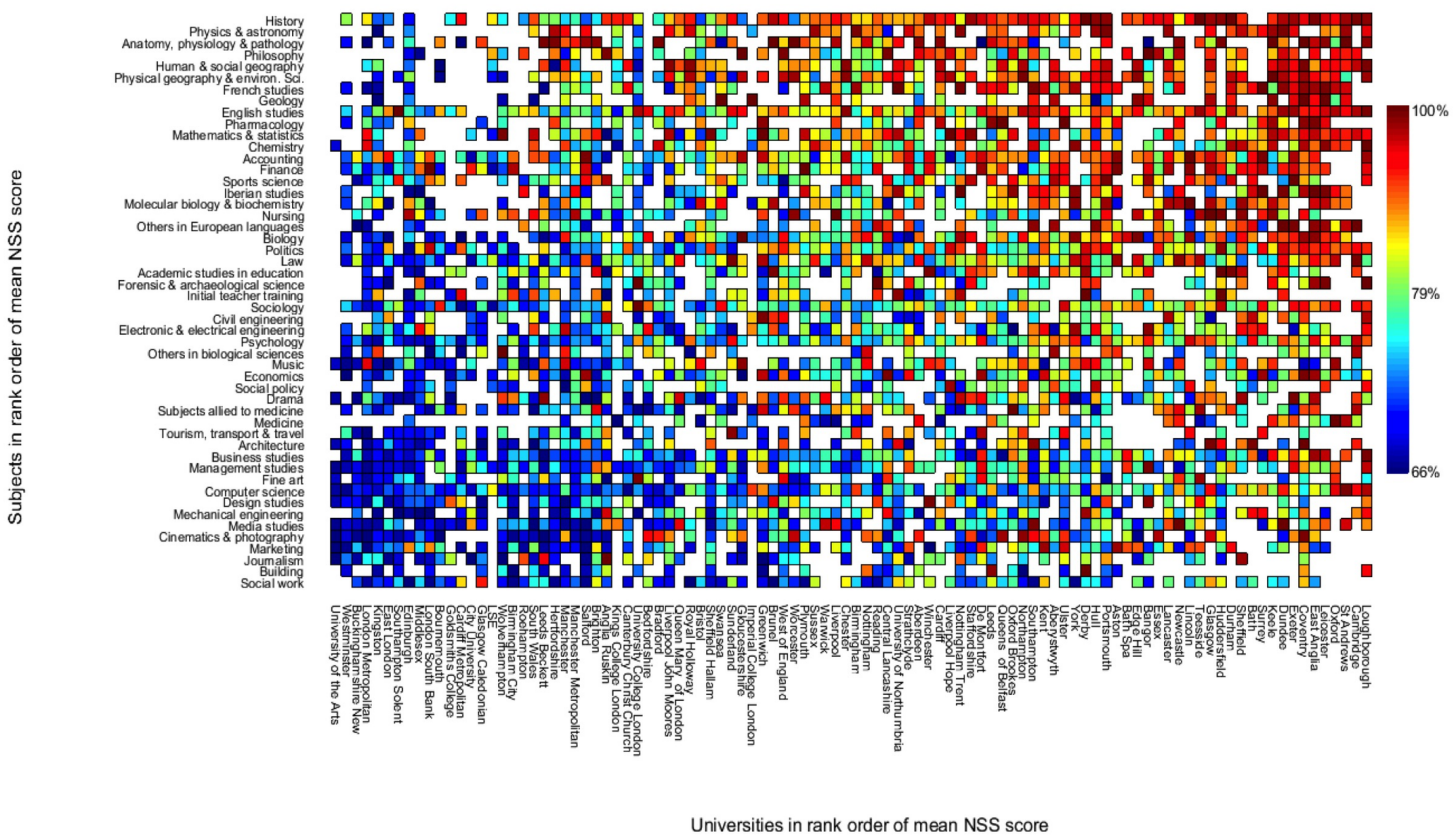
Heat map showing the mean NSS satisfaction score (averaged over items 1 to 22 in the decade 2007–2016) for the largest 100 HEIs and the 50 most popular subject areas in the UKHE sector. White squares indicate subjects that are not taught at that university. HEIs and subjects are presented in rank order of their mean NSS scores.

Fig 3 shows showing the mean NSS satisfaction score (averaged over items 1 to 22 in the decade 2007–2016) for the largest 100 HEIs and the 50 most popular subject areas in the UKHE sector with the HEIs and subjects in rank order of their mean NSS scores. There was a tendency for the sciences and humanities to receive higher satisfaction ratings than social and creative disciplines and there was a noticeable trend for research intensive universities (The ‘Russell Group’ and the ‘1994 Group’) to perform better than teaching focussed institutions (The ‘Million+ group’).

### Predictors of overall student satisfaction

In order to determine the relative importance of the different components of the NSS in predicting overall satisfaction, the factors generated from the factor analysis from data from all years combined were entered as predictor variables into a regression analysis with item 22, ‘Overall Satisfaction’, as the dependent variable. The resultant model significantly predicted ‘Overall Satisfaction’ (F_6, 33.3_ = 17.9, p < .001; R = 0.87, adjusted R^2^ = 0.76). The analysis was then repeated for each year separately and in each case the factor scores significantly predicted ‘Overall Satisfaction’ with the adjusted R^2^ varying between 0.74 and 0.80 (see Table 1). Table 1 also shows that the strongest predictors of ‘Overall Satisfaction’ were ‘Organisation & Management’ (overall β = .52) and ‘Teaching Quality’ (overall β = .49) with these factors alone accounting for 57% of the variance. The β-weights were fairly stable over the decade except that the importance of ‘Teaching Quality’ tended to increase from 2007–2016 whilst the importance of ‘Organisation & Management’ and ‘Academic Support’ both showed small decreases. The other factors showed no consistent direction of change. ‘Assessment & Feedback’ was a relatively poor predictor (β = 0.18); its contribution was weaker than that of ‘Personal Development’ (β = 0.30).

**Table 1:** NSS predictors of ‘Overall Satisfaction’ showing the standardised Beta coefficients for each of the six NSS subscales for each year from 2007 to 2016.

Factor	2007	2008	2009	2010	2011	2012	2013	2014	2015	2016	All Years
**Teaching**	0.44	0.43	0.45	0.48	0.49	0.49	0.49	0.51	0.46	0.49	0.49
**Assessment & Feedback**	0.19	0.19	0.18	0.18	0.19	0.16	0.17	0.20	0.23	0.20	0.18
**Academic Support**	0.27	0.25	0.23	0.21	0.20	0.21	0.23	0.20	0.17	0.17	0.18
**Organisation & Management**	0.55	0.58	0.53	0.52	0.52	0.49	0.49	0.49	0.47	0.48	0.52
**Learning Resources**	0.13	0.12	0.18	0.15	0.15	0.15	0.13	0.11	0.14	0.13	0.14
**Personal Development**	0.25	0.29	0.29	0.31	0.33	0.31	0.30	0.31	0.29	0.30	0.30
**Adjusted R**^**2**^	0.76	0.80	0.78	0.80	0.80	0.76	0.76	0.78	0.73	0.74	0.76

### The effects of increases in tuition fees

**Fig 4:**
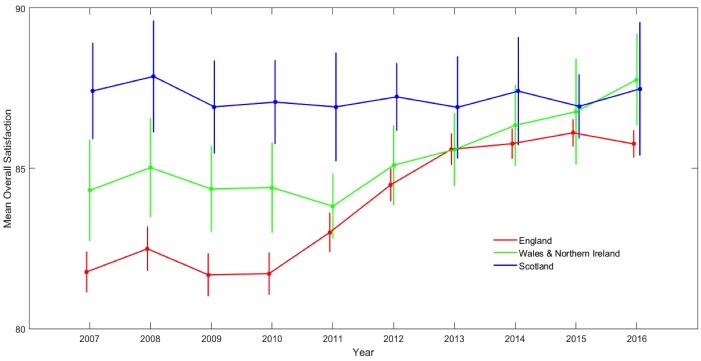
Mean ‘Overall Satisfaction’ for the devolved national undergraduate cohorts (England = high tuition fees; Wales and Northern Ireland Intermediate tuition fees; Scotland. Free tuition fees). Error bars indicate ±1 standard errors from the mean. Tuition fees were payable throughout the period (except in Scotland) and were increased in 2012, affecting the NSS cohort for the first time in 2015.

The mean ‘Overall Satisfaction’ scores by fee level (England = ‘high’, n = 85; Northern Ireland and Wales combined = ‘intermediate’, n = 8; Scotland = ‘none’, n = 7) for each year 2007 to 2016 are shown in Fig 4. In 2007, students in Scotland reported the highest levels of ‘Overall Satisfaction’ (87.4%) followed by those in Northern Ireland & Wales (84.3%) and then England (81.8%). This appears to support an inverse association between tuition fees and overall student satisfaction except that, at that time, students in England, Wales and Northern Ireland were all paying the same level of fees. Furthermore, changes in student satisfaction thereafter do not appear to support any such association. For students attending Scottish Universities, ‘Overall Satisfaction’ has remained steady across the decade which is what might be expected given that Scottish students have experienced no change in tuition fees during this period. However, ‘Overall Satisfaction’ for students attending English Universities, which started well below Scottish levels in 2007, increased steadily thereafter and achieved parity from 2013 onwards. Most strikingly, the tripling of fees in 2012, which would first be paid by students completing the NSS in 2015, appears to have had no effect on ‘Overall Satisfaction’ at all. Students in Wales and Northern Ireland showed a similar trend to those in England but started off the decade with slightly higher levels of ‘Overall Satisfaction’. In short, although there have been changes in student satisfaction over the decade, at least outside Scotland, these changes do not appear to be related to changes in tuition fees.

## Discussion

### Helping universities improve

Our analysis of the student satisfaction in UK higher education in the period 2007–2016 reveals a story of success and stability. Success, because student satisfaction on all NSS subscales has improved across the decade (with the lowest rated items in 2007 showing the most improvement) which is a powerful confirmation of the value of the NSS and shows that universities have responded successfully to the feedback that the NSS provided. Stability, because the factors that influence NSS ratings have, with some important exceptions, remained unchanged.

### Helping prospective students choose

The information provided by the NSS to help prospective students choose the right university for their chosen subject has actually improved with time. In their description of the original development of the NSS Richardson *et*. *al* [29] noted that the ‘University’ explained 7% of variance and the ‘Subject’ explained 7%, whereas the interaction between ‘University’ and ‘Subject’ explained 24%, confirming the validity of the scale to discriminate between different institutions offering the same subject. Our results from 2007 suggest a similar proportion of variance for ‘University’ (7.5%) and the ‘University x Subject’ interaction (20.9%) but rather less for ‘Subject’ (4%). Intriguingly, however, this is one area where the NSS has revealed evidence of change. By 2016, the role of ‘University’ had increased to 11.6% (an increase of 55%) and the ‘University x Subject’ component had increased to 27.6% (an increase of 32%). This latter finding suggests that the information provided by the NSS to help prospective students choose the right university for their chosen subject has actually improved with time. Yorke *et al*. [33] have previously discussed the relatively small contribution of ‘Subject’ to student satisfaction with a particular focus on the generally lower scores experienced by Art and Design programmes, a finding that we confirm. They argued that the nature of some of the questions may disadvantage certain types of programme and warned against coalescing NSS ratings from all the subject areas which *“implicitly treat differences between subject areas as being of little consequence”*. Gibbs ([38], p.46*)* argued that: *“either one has to accept that certain subjects are always taught less well than others*, *which seems highly unlikely*, *or that different measures of quality are better aligned with the consequences of some (disciplinary) pedagogic practices than with others”*. We would only add that, whatever the role of ‘Subject’ in student satisfaction, it appears to be diminishing.

Our finding that ‘University’ accounts for only a small proportion of the total variance in NSS scores is also consistent with previous studies. Both Surridge [39] and Cheng and Marsh [39] found that although the differences between institutions was small, it was statistically reliable. However, they go further and suggest that individual student characteristics such as ethnicity can also explain much of the variance explained by universities (see also [40]) and posit that relatively few universities actually differ significantly from the mean of all universities.

### Predictors of overall student satisfaction

Our analyses of the best predictors of ‘Overall Satisfaction’ tally with Langan *et al*. [41] who found that best ‘thematic predictors’ of overall satisfaction were ‘Teaching Quality’ and ‘Organisation & Management’, but the single best predictor of ‘Overall Satisfaction’ was “*The course was well designed and running smoothly*”. Langan *et al*. [42] extended this analysis further and performed a textual analysis of the open comments section of the NSS which also suggested course organisation as a key moderating factor on satisfaction. Given that teaching scores are relatively high across the sector, and may be close to ceiling for many institutions, HEIs may be best advised to concentrate on course organisation as a way to improve their NSS scores. This is particularly relevant in the light of Fielding *et al*.’s [43] argument that support and organisation may be an institutional-level issue rather than a subject level issue, meaning that HEIs have the potential to take action to improve these scores across the board. In this context is it worth remembering Agnew *et al*.’s [37] finding that ‘Organisation & Management’ was positively correlated with staff-student ratio. Most important of all, HEIs need to beware of ‘critical incidents’ that can disproportionately influence responses so it would be important to avoid any of these types of hiccups around the NSS period [44].

In reality, universities seem to have focussed most of their efforts over the decade on improving the lowest scoring components of the NSS (most commonly ‘Assessment & Feedback’), a temptation that Fielding *et al*. [43] acknowledge seems beneficial. They warn, however, that the relationship between ‘Assessment & Feedback’ and ‘Overall Satisfaction’ is complex and differs across subject groups making it more difficult to correct. In addition, as we have shown, ‘Assessment & Feedback’ was a surprisingly weak predictor of overall satisfaction meaning that even a very large success in improving ‘Assessment & Feedback’ would have only modest effects on ‘Overall Satisfaction’.

Fielding *et al*. [43] also found that ‘Learning Resources’ had at most weak correlations with ‘Overall Satisfaction’, a finding that we confirm, and appeared not to differentiate institutions or subjects. This suggests that large scale investments, for example in building projects and refurbishments, may not be particularly effective in improving student satisfaction. This observation is supported by Agnew *et al*. [37] who found that neither learning resources nor spend-per-student correlated with satisfaction levels in business schools. Of course, even if these large scale investments have only a marginal role in influencing student satisfaction, they may be useful in attracting students to the university in the first place.

The effects of increases in tuition feesThe burden of student debt on recent undergraduates has become an increasingly sensitive political topic and was a divisive issue in the UK’s 2017 General Election where a surge in support for the opposition party from younger voters was attributed, in large part, to their promise to abolish tuition fees. Despite this, there appears to be no obvious effect of tuition fees on NSS results. Although English students (high fees) and Welsh and Northern Irish students (intermediate fees) showed lower levels of student satisfaction at the start of the decade than Scottish students (no fees), these differences had closed by 2016, despite the tripling of fees for the English.

The fact that increased fees have coincided with increased student satisfaction appears counter-intuitive and requires explanation, but it should be noted that inversions of conventional market relationships are not without precedent. It might be that higher education is a Veblen good and that students value their education, at least in part, because it is perceived as being expensive. One might view this as a case of cognitive dissonance [45] and argue that students cope with the experience of having to pay increased fees by up-rating the value they place on their education. However, as the NSS does not directly ask about ‘value for money’ or other cost-related issues, its insensitivity to fee increases should perhaps be expected. Students who completed the NSS had for the most part, not yet had to pay the fees themselves; most would have taken out a loan that might not need to be repaid for several years and in some cases would never need to be paid. A follow-up of graduates in the process of repaying their loans might give a different view of student satisfaction. Whatever the reason, the insensitivity of the NSS to the costs of higher education seems to be an important omission and one that could be readily redressed.

### Limitations

Throughout this study, we have used the mean scores for each university subject group and in doing so, we have treated all university subject groups with equal weighting when, in reality, they consisted of different numbers of students. Our reason for doing this is that the focus of our interest was on the factors that contributed to student satisfaction for university subject groups themselves, that might be useful for strategic judgements, rather than on the factors influencing individual students. Nevertheless, the fact that the largest proportion of variance in NSS scores remains unaccounted for suggests there is more we might learn from considering the individual more fully. For example, we did not take into account the sex, age or ethnicity of students, factors known to influence reported satisfaction levels on the NSS [29].

There are, of course, many other factors which are likely to influence the ‘Overall Satisfaction’ measure in the NSS which are not actually measured by it and factors which are measured by it which we did not include in our analyses. Lenton [46] suggested that university type (possibly as a mediator of perceived graduate outcomes), degree outcome, employment outcomes and staff student ratio all play a role. Richardson *et al*. [29] listed categories of comments in the open text section of the NSS most commonly received in the development of the NSS and subsequent to comments regarding ‘quality of teaching’ (12.7%) and ‘level of support’ (12.0%) came ‘social life, meeting people and accommodation’ (which represented 9.8% of the comments). Comments also included: ‘organisation’ (8.2%); ‘useful/ relevant to my job’ (5.7%); ‘option choices’ (6.6%); ‘feedback’ (5.7%); ‘library’ (5.3%); and ‘workload’ (5.0%). Douglas *et al*. [47] analysed narratives from 350 students using qualitative techniques and suggested that important determinants of quality were ‘Access’, ‘Attentiveness’, ‘Availability’ and ‘Communication’ whereas Wiers-Jenssen *et al*. [48] concluded that ‘social climate’, ‘aesthetic aspects of the physical infrastructure’ and ‘quality of administration’ all contribute to the student experience. Brown *et al*. [49] suggest that student evaluations, for example of satisfaction with promptness of feedback, may be highly influenced by their beliefs about what happens elsewhere and argue that more objective measures of time should be substituted. Clearly, the role of the individual student in judgments of satisfaction is more complex and varied that we have been able to address in this paper.

## Conclusion

There is good evidence that student satisfaction has improved substantially over the last decade and that those areas of greatest weakness (e.g. ‘Assessment & Feedback’) have shown greatest improvement. The NSS has proved to be a robust and stable tool over the last decade and, in as much as it has changed, it has improved and now offers better discrimination between courses at different universities than it did before. For the educational manager who wants to improve their institutional ratings, it is clear that ‘Teaching Quality’ and ‘Organisation & Management’ are the most important factors in shaping student satisfaction and, as teaching quality appears to be near ceiling levels, a ruthless emphasis on the smooth running and good design of courses would seem to be a sensible route to follow. If the NSS has one limitation, it is that it fails to address student perception of value-for-money and, with the increasing influence of market forces in HE, this seems to be an important oversight.

## Supporting information

S1 Table List of the 100 universities included in the study. (PDF)

S2 Table List of the level 3 subject groups used in the NSS. (PDF)

S3 Table Principal axis factor loadings of the 21 NSS items (excluding ‘Overall Satisfaction’) on the six factors, corresponding to the 6 subscales of the NSS, for the combined NSS data 2007–2016. (PDF)

S1 File A brief history of tuition fees in the UK since 1962. (PDF)
