# Supplementary material for: A 10-year case study on the changing determinants of university student satisfaction in the UK
Source: PLoS One. 2018 Feb 23;13(2):e0192976. doi: 10.1371/journal.pone.0192976 (PMC5825039; doi:10.1371/journal.pone.0192976)
Supplement: S1 Table — (PDF) [file pone.0192976.s001.pdf]

S1 Table. List of the 100 universities included in the study

UKPRN	Higher Education Institution (HEI)	HEI Group	Nation	Fee Level
10007783	Aberdeen	-	Scotland	Free
10007856	Aberystwyth	-	Wales	Intermediate
10000291	Anglia Ruskin	Million+ Group	England	High
10007759	Aston	-	England	High
10007857	Bangor	-	Wales	Intermediate
10007850	Bath	-	England	High
10000571	Bath Spa	Million+ Group	England	High
10007152	Bedfordshire	Million+ Group	England	High
10006840	Birmingham	Russell Group	England	High
10007140	Birmingham City	-	England	High
10000824	Bournemouth		England	High
10007785	Bradford	-	England	High
10000886	Brighton	University Alliance	England	High
10007786	Bristol	Russell Group	England	High
10000961	Brunel	-	England	High
10000975	Buckinghamshire New	-	England	High
10007788	Cambridge	Russell Group	England	High
10001143	Canterbury Christ Church	Million+ Group	England	High
10007814	Cardiff	Russell Group	Wales	Intermediate
10007854	Cardiff Metropolitan	-	Wales	Intermediate
10007141	Central Lancashire	University Alliance	England	High
10007848	Chester	-	England	High
10001478	City University	-	England	High
10001726	Coventry	University Alliance	England	High
10001883	De Montfort	-	England	High
10007851	Derby	-	England	High
10007852	Dundee	-	Scotland	Free
10007143	Durham	Russell Group	England	High
10007789	East Anglia	1994 Group	England	High
10007144	East London	Million+ Group	England	High
10007823	Edge Hill	-	England	High
10007790	Edinburgh	Russell Group	Scotland	Free
10007791	Essex	1994 Group	England	High
10007792	Exeter	Russell Group	England	High
10007794	Glasgow	Russell Group	Scotland	Free
10007762	Glasgow Caledonian	-	Scotland	Free
10007145	Gloucestershire	-	England	High
10002718	Goldsmiths College	1994 Group	England	High
10007146	Greenwich	University Alliance	England	High
10007147	Hertfordshire	University Alliance	England	High
10007148	Huddersfield	University Alliance	England	High

10007149	Hull	-	England	High
10003270	Imperial College London	Russell Group	England	High
10007767	Keele	-	England	High
10007150	Kent	-	England	High
10003645	Kings College London	Russell Group	England	High
10003678	Kingston	University Alliance	England	High
10007768	Lancaster	1994 Group	England	High
10007795	Leeds	Russell Group	England	High
10003861	Leeds Beckett	-	England	High
10007796	Leicester	1994 Group	England	High
10007151	Lincoln	University Alliance	England	High
10003956	Liverpool Hope	-	England	High
10003957	Liverpool John Moores	University Alliance	England	High
10006842	Liverpool	Russell Group	England	High
10004048	London Metropolitan	Million+ Group	England	High
10004078	London South Bank	Million+ Group	England	High
10004113	Loughborough	1994 Group	England	High
10004063	LSE	Russell Group	England	High
10007798	Manchester	Russell Group	England	High
10004180	Manchester Metropolitan	University Alliance	England	High
10004351	Middlesex	Million+ Group	England	High
10007799	Newcastle	Russell Group	England	High
10007138	Northampton	-	England	High
10007154	Nottingham	Russell Group	England	High
10004797	Nottingham Trent	University Alliance	England	High
10007774	Oxford	Russell Group	England	High
10004930	Oxford Brookes	University Alliance	England	High
10007801	Plymouth	University Alliance	England	High
10007155	Portsmouth	University Alliance	England	High
10007775	Queen Mary of London	Russell Group	England	High
10005343	Queens of Belfast	Russell Group	N. Ireland	Intermediate
10007802	Reading	-	England	High
10007776	Roehampton	-	England	High
10005553	Royal Holloway	1994 Group	England	High
10007156	Salford	University Alliance	England	High
10007157	Sheffield	Russell Group	England	High
10005790	Sheffield Hallam	University Alliance	England	High
10007793	South Wales	University Alliance	Wales	Intermediate
10007158	Southampton	Russell Group	England	High
10006022	Southampton Solent	Million+ Group	England	High
10007803	St Andrews	-	Scotland	Free
10006299	Staffordshire	Million+ Group	England	High
10007805	Strathclyde	-	Scotland	Free
10007159	Sunderland	Million+ Group	England	High

10007160	Surrey	-	England	High
10007806	Sussex	1994 Group	England	High
10007855	Swansea	-	Wales	Intermediate
10007161	Teesside	University Alliance	England	High
10007807	Ulster	-	N. Ireland	Intermediate
10007784	University College London	Russell Group	England	High
10001282	University of Northumbria	-	England	High
10007162	University of the Arts	-	England	High
10007163	Warwick	Russell Group	England	High
10007164	West of England	University Alliance	England	High
10007165	Westminster	-	England	High
10003614	Winchester	-	England	High
10007166	Wolverhampton	-	England	High
10007139	Worcester	-	England	High
10007167	York	Russell Group	England	High
