# Supplementary material for: A 10-year case study on the changing determinants of university student satisfaction in the UK
Source: PLoS One. 2018 Feb 23;13(2):e0192976. doi: 10.1371/journal.pone.0192976 (PMC5825039; doi:10.1371/journal.pone.0192976)
Supplement: S2 Table — (PDF) [file pone.0192976.s002.pdf]

S2 Table. List of the Level 3 Subject Groups used in the NSS

Level 3 Code	Subject
1	'Medicine'
2	'Dentistry'
3	'Anatomy, physiology & pathology'
4	'Pharmacology'
5	'Nursing'
6	'Complementary medicine'
7	'Nutrition'
8	'Ophthalmics'
9	'Aural & oral sciences'
10	'Medical technology'
11	'Subjects allied to medicine'
12	'Biology'
13	'Zoology'
14	'Genetics'
15	'Microbiology'
16	'Molecular biology & biochemistry'
17	'Others in biological sciences'
18	'Sports science'
19	'Psychology'
20	'Veterinary sciences'
21	'Animal science'
22	'Forestry'
23	'Food & beverage studies'
24	'Agriculture & related subjects'
25	'Chemistry'
26	'Physics & astronomy'
27	'Forensic & archaeological science'
28	'Geology'
29	'Ocean sciences'
30	'Others in physical sciences'
31	'Physical geography & environ. Sci.'
32	'Mathematics & statistics'
33	'Operational research'
34	'Other Maths '
35	'Computer science'
36	'General engineering'
37	'Mechanical engineering'
38	'Aerospace engineering'
39	'Naval architecture'
40	'Electronic & electrical engineering'

41	'Civil engineering'
42	'Chemical engineering'
43	'Others in engineering'
44	'Materials & minerals technology'
45	'Maritime technology'
46	'Others in technology'
47	'Architecture'
48	'Building'
49	'Landscape design'
50	'Planning (urban, rural & regional)'
51	'Architecture, building & planning'
52	'Economics'
53	'Politics'
54	'Sociology'
55	'Social policy'
56	'Anthropology'
57	'Others in social studies'
58	'Social work'
59	'Human & social geography'
60	'Law'
61	'Business studies'
62	'Marketing'
63	'Management studies'
64	'Human resource management'
65	'Finance'
66	'Accounting'
67	'Tourism, transport & travel'
68	'Business & administrative studies'
69	'Media studies'
70	'Information services'
71	'Publicity studies'
72	'Publishing'
73	'Journalism'
74	'Mass communications '
75	'English studies'
76	'American & Australasian studies'
77	'Celtic studies'
78	'Classics'
79	'French studies'
80	'German & Scandinavian studies'
81	'Italian studies'
82	'Iberian studies'
83	'Others in European languages'
84	'Linguistics'

85	'Comparative literary studies'
86	'Linguistics, classics & related'
87	'Asian studies'
88	'African & Middle Eastern studies'
89	'Eastern, Asian & African languages'
90	'History'
91	'Archaeology'
92	'Historical & philosophical studies'
93	'Philosophy'
94	'Theology & religious studies'
95	'Fine art'
96	'Design studies'
97	'Music'
98	'Drama'
99	'Dance'
100	'Cinematics & photography'
101	'Imaginative writing'
102	'Others in creative arts & design'
103	'Teacher training'
104	'Research & study skills in education'
105	'Academic studies in education'
106	'Others in education'
107	'Combined'
108	'Initial teacher training'
