# Supplementary material for: A 10-year case study on the changing determinants of university student satisfaction in the UK
Source: PLoS One. 2018 Feb 23;13(2):e0192976. doi: 10.1371/journal.pone.0192976 (PMC5825039; doi:10.1371/journal.pone.0192976)
Supplement: S3 Table — (PDF) [file pone.0192976.s003.pdf]

## S3 Table. Factor Loadings of the NSS items on the Six Factors

| NSS Item | Putative NSS Category | Communalities | Factor Number | | | | | |
|---|---|---|---|---|---|---|---|---|
| | | | 1 | 2 | 3 | 4 | 5 | 6 |
| Q1 | Teaching Quality | 0.69 | 0.22 | **0.63** | 0.40 | 0.21 | 0.07 | 0.21 |
| Q2 | | 0.84 | 0.28 | **0.82** | 0.16 | 0.25 | 0.02 | 0.05 |
| Q3 | | 0.73 | 0.31 | **0.73** | 0.23 | 0.19 | 0.07 | 0.12 |
| Q4 | | 0.64 | 0.09 | **0.67** | 0.34 | 0.21 | 0.14 | 0.10 |
| Q5 | Assessment & Feedback | 0.47 | **0.53** | 0.06 | 0.30 | 0.26 | 0.07 | 0.17 |
| Q6 | | 0.51 | **0.45** | 0.25 | 0.39 | 0.11 | 0.14 | 0.24 |
| Q7 | | 0.61 | **0.70** | 0.15 | 0.26 | 0.10 | 0.12 | 0.11 |
| Q8 | | 0.84 | **0.87** | 0.23 | 0.06 | 0.12 | -0.08 | -0.01 |
| Q9 | | 0.80 | **0.83** | 0.22 | 0.11 | 0.18 | 0.08 | 0.10 |
| Q10 | Academic Support | 0.81 | 0.42 | 0.37 | 0.27 | 0.33 | 0.18 | **0.53** |
| Q11 | | 0.60 | 0.20 | 0.38 | **0.43** | 0.13 | 0.22 | 0.41 |
| Q12 | | 0.74 | 0.42 | 0.31 | 0.26 | 0.32 | 0.22 | **0.50** |
| Q13 | Organisation & Management | 0.45 | 0.14 | 0.21 | **0.61** | 0.08 | 0.09 | 0.01 |
| Q14 | | 0.81 | 0.20 | 0.17 | **0.84** | 0.09 | 0.16 | 0.11 |
| Q15 | | 0.87 | 0.20 | 0.27 | **0.85** | 0.10 | 0.11 | 0.13 |
| Q16 | Learning Resources | 0.56 | 0.04 | 0.01 | 0.09 | 0.10 | **0.73** | 0.05 |
| Q17 | | 0.67 | 0.04 | 0.07 | 0.07 | 0.07 | **0.81** | 0.02 |
| Q18 | | 0.68 | 0.09 | 0.12 | 0.18 | 0.20 | **0.76** | 0.14 |
| Q19 | Personal Development | 0.82 | 0.21 | 0.23 | 0.12 | **0.82** | 0.14 | 0.11 |
| Q20 | | 0.77 | 0.16 | 0.18 | 0.04 | **0.84** | 0.11 | 0.03 |
| Q21 | | 0.73 | 0.18 | 0.24 | 0.17 | **0.73** | 0.22 | 0.16 |

The highest loading for each item is shown in bold
