# Supplementary material for: A 10-year case study on the changing determinants of university student satisfaction in the UK
Source: PLoS One. 2018 Feb 23;13(2):e0192976. doi: 10.1371/journal.pone.0192976 (PMC5825039; doi:10.1371/journal.pone.0192976)
Supplement: S1 File — (PDF) [file pone.0192976.s004.pdf]

S1 File. A Brief History of Tuition Fees in the UK since 1962[1]

In the UK, successive governments have responded to the increasing costs of Higher Education (HE) by introducing market forces to the sector and by transferring the cost from the state to individual students. University education in the UK was free from 1962 until tuition fees were re-introduced in 1998. However, education is devolved in the UK which has allowed England, Northern Ireland, Scotland and Wales to adopt very different policies with regards to university tuition fees over the last decade. In consequence, the UK HE sector presents an excellent opportunity to track the effects of the changing costs of university education on student satisfaction.

England

In England, students were required to pay variable annual tuition fees of anything up to £1,000 from 1998 and up to £3,000 from 2006. From 2012, following the 'Browne Report' (Browne, 2010), students took on the full cost of their education and tuition fees were increased to £9,000.

Wales

Welsh students, for example, are eligible for the same fees as English students but, from 2002 onwards they have received a grant to help cover their tuition fees. Initially, means tested, this grant was £1,500 in 2002, increasing to £1,989, when tuition fees were capped at £3,000 in 2006 and £4,954 following the increased cap on fees of £9,000 in 2016/7 and no longer means-tested. Uniquely, Welsh students can use this grant to cover tuition fees charged in universities outside of Wales which allows them to study anywhere in the UK for £4,046. Universities in Wales have their fees capped at the same level as England (£9,000) and UK students from outside the principality are eligible to pay them in full.

Northern Ireland

Northern Ireland followed the same path as England until (up to £3,000 from 2006-2011) but has since adopted a policy of reduced fees and currently charges £3,805 for home students and the full £9,000 for students from other parts of the UK.

Scotland

Scotland has taken a very different path. Until 2007, Scottish students attending Scottish Universities initially paid a one-off 'endowment' fee of £2,289 at the end of their degree (typically 4 years in Scotland) but this was abolished in 2007 and since then, students have

---

[1] Costs are based on fees up to the academic year 2016/17 which is the last year covered by this study. Tuition fees in the UK increase in 2017/18 and are now capped at £9250

received free tuition. However, students from other parts of the UK attending Scottish universities pay up to £9,000.
